# Predictive value of coronary artery calcification for osteoporosis in COPD patients: a retrospective AI-based study

**DOI:** 10.3389/fmed.2026.1799838

**Published:** 2026-07-07

**Authors:** Lijin He, Fan Meng, Luting Zhang, Yangli Zheng, Zhishuang Song, Meifang Li

**Affiliations:** 1 Department of Radiology, Affiliated Hospital of Putian University, Putian, Fujian, China; 2 College of Biomedical Engineering and Instrument Science, Zhejiang University, Hangzhou, China; 3 Department of Medical Ultrasonics, Fujian Maternity and Child Health Hospital, College of Clinical Medicine for Obstetrics and Gynecology and Pediatrics, Fujian Medical University, Fuzhou, China; 4 Department of Pediatric Critical Care Medicine, Affiliated Hospital of Putian University, Putian, Fujian, China

**Keywords:** artificial intelligence, chronic obstructive pulmonary disease, computed tomography, coronary artery calcification, osteoporosis

## Abstract

**Background:**

Patients with chronic obstructive pulmonary disease (COPD) are at high risk for osteoporosis (OP), yet OP screening remains suboptimal in practice. The “bone–vascular axis” hypothesis suggests shared mechanisms between vascular calcification and bone loss. Coronary artery calcification (CAC), a reliable imaging marker of atherosclerosis, may serve as a surrogate indicator of OP risk in this population. Advances in artificial intelligence (AI) now enable automated CAC quantification from routine chest CT scans, offering a potentially efficient, low-cost strategy for opportunistic OP risk assessment in COPD patients.

**Objective:**

This study aimed to evaluate the predictive value of artificial intelligence (AI)-based CAC scoring, derived from routine chest computed tomography (CT) scans, for identifying osteoporosis in patients with COPD.

**Methods:**

The retrospective study included COPD patients who underwent dual-energy X-ray absorptiometry (DXA) and non-gated chest CT between 2020 and 2025. Osteoporosis was defined as a T-score ≤ -2.5. An AI tool automatically quantified Agatston scores for all four coronary arteries. Statistical analyses involved intergroup comparisons and adjusted logistic regression, with ROC curves assessing predictive performance.

**Results:**

Amongst the 262 patients, 117 were diagnosed with osteoporosis. The osteoporosis group was characterized by older age, lower BMI, and greater coronary artery calcification scores in all arteries. Following adjustment for confounding factors including age, sex, and BMI, left anterior descending coronary artery calcification (LAD-CAC; OR = 1.01, 95% CI: 1.00–1.02, *P* = 0.036) and left circumflex coronary artery calcification (LCX-CAC; OR = 1.11, 95% CI: 1.02–1.20, *P* = 0.021) were identified as independent predictors of osteoporosis. The area under the curve (AUC) values were 0.703 (sensitivity 51.3%, specificity 87.6%) and 0.625 (sensitivity 31.6%, specificity 93.1%), respectively.

**Conclusion:**

Coronary artery calcium scores, particularly LAD-CAC and LCX-CAC, automatically obtained via AI software from routine chest CT scans, are independently associated with osteoporosis in COPD patients and demonstrate moderate predictive capability. This finding suggests a predictive utility for early, convenient, and cost-free screening of OP risk by leveraging routine imaging examinations already performed in COPD patients.

## 1 Introduction

Chronic obstructive pulmonary disease (COPD) is a leading cause of global mortality, with an estimated prevalence of approximately 10.6% among adults aged 40 years and older (1). Beyond impaired lung function, COPD is characterized by significant systemic complications, among which osteoporosis (OP) is particularly salient. Fragility fractures in these patients impose severe restrictions on thoracic mobility, exacerbate respiratory failure, and instigate a detrimental cycle that substantially elevates morbidity and mortality (2, 3). Despite the evident risks, osteoporosis remains considerably underdiagnosed in the COPD population, primarily due to limited clinical access to the gold standard diagnostic method—dual-energy X-ray absorptiometry (DXA) (4). Consequently, there is an urgent need to develop efficient and accessible screening tools to be seamlessly integrated into routine COPD clinical pathways.

The conceptual framework of the “bone-vascular axis” provides a robust physiological rationale for this study. Coronary artery calcification (CAC) has been established as a reliable and quantifiable CT marker of subclinical atherosclerosis (5). Emerging evidence suggests that CAC and bone metabolism disorders may share common pathophysiological pathways, including chronic inflammation and dysregulation of the RANKL/OPG signaling pathway (6, 7). However, the association between CAC and bone mineral density in the general population remains inconsistent (3, 8, 9), with particularly scarce data in high-risk groups such as those with COPD.

Concurrent advancements in medical image analysis have yielded promising solutions. The advent of artificial intelligence (AI) technologies, particularly deep learning, has enabled the automated and precise quantification of CAC from non-gated chest CT scans. These scans are now a standard component of routine COPD management, facilitating consistent patient monitoring (10). This capability presents a unique opportunity for the “opportunistic utilization of existing imaging data, allowing for the extraction of potential osteoporosis risk biomarkers without imposing additional radiation or financial burdens on patients.”

The impetus for this study was driven by both clinical demand and technological feasibility. The objective was to validate whether AI- derived CAC scores from standard chest CT scans can independently predict osteoporosis in patients with COPD. It is hypothesized that calcification within specific coronary regions possesses independent predictive value and may serve as a practical screening indicator. If validated, this approach could offer an innovative integrated strategy for the comprehensive management of COPD.

## 2 Materials and methods

### 2.1 Study design and subject selection

**Figure 1:**
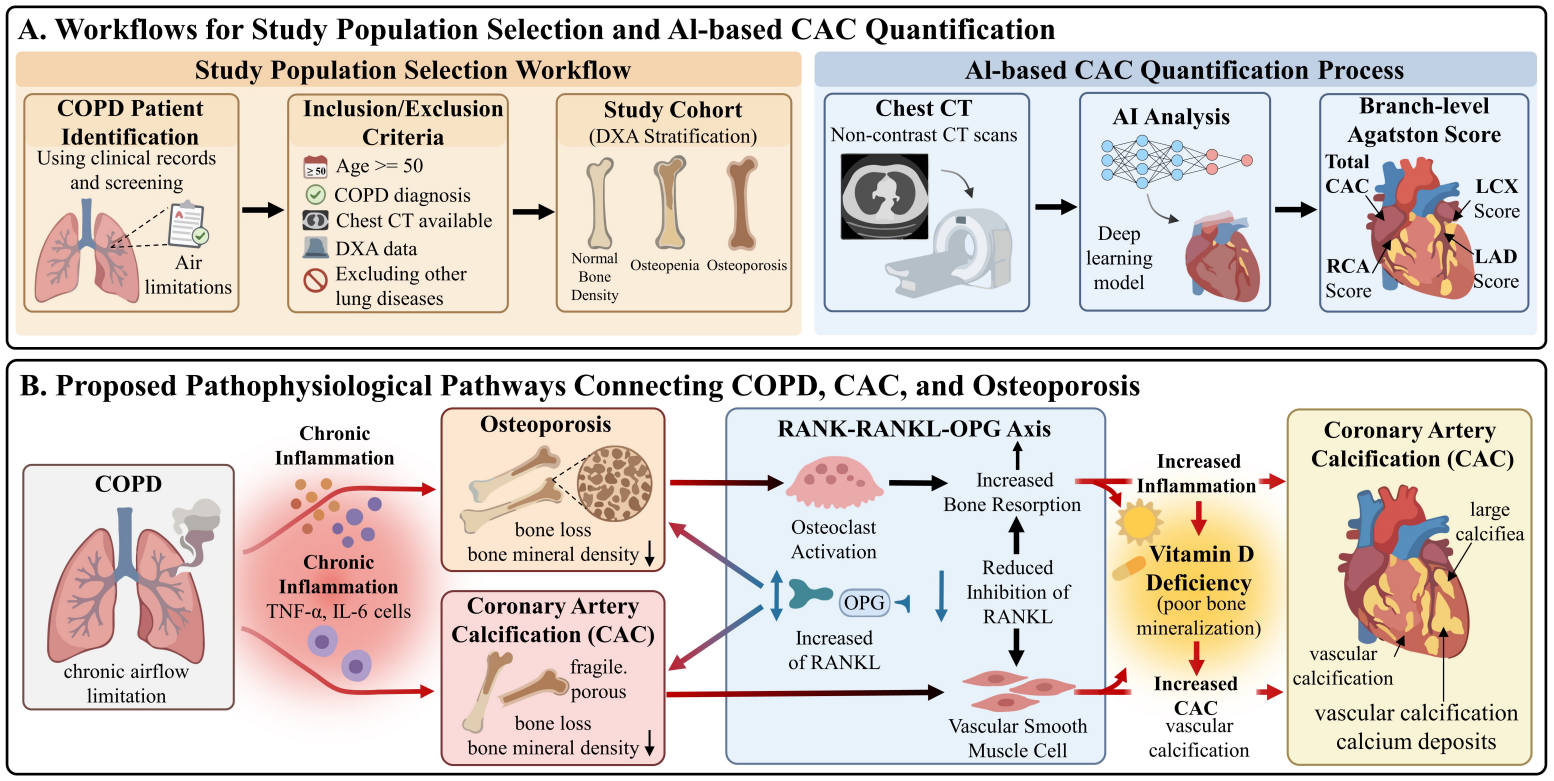
Visual abstract illustrating: **(A)** study patient selection workflow with inclusion/exclusion criteria and DXA-based outcome stratification; **(B)** the AI-based coronary artery calcium (CAC) quantification pipeline using non-gated chest CT; and proposed shared pathophysiological mechanisms linking COPD, vascular calcification, and osteoporosis via the RANK-RANKL-OPG axis, chronic systemic inflammation, and vitamin D deficiency.

This retrospective study analyzed clinical and imaging records of patients diagnosed with COPD at our hospital between January 2020 and November 2025. COPD was diagnosed according to current Global Initiative for Chronic Obstructive Lung Disease (GOLD) guidelines, including a FEV1/FVC ratio of less than 70% following a bronchodilator (11). The diagnosis of OP was made using the WHO criteria. This diagnosis was determined through the implementation of a DXA scan, a procedure that quantifies the mineral density of the patient's bones. The specific regions of the patient's body that were assessed included the lumbar spine and the femoral neck. A T-score ≤ -2.5 standard deviations at any measurement site was defined as osteoporosis (12). The cohort was stratified into two mutually exclusive groups based on DXA-measured T-scores for comparative analysis: COPD with concomitant OP and the COPD without OP (T-score > −2.5; Figures 1A, B).

### 2.2 Inclusion and exclusion criteria

#### 2.2.1 Inclusion criteria

(1) Meet the aforementioned diagnostic criteria for chronic obstructive pulmonary disease (COPD); (2) be aged ≥50 years at the time of evaluation; (3) complete pulmonary function testing, non-contrast chest CT scan, and dual-energy X-ray absorptiometry (DXA) within standard clinical timeframes; (4) technically adequate chest CT images for reliable quantitative analysis, with DXA and non-gated chest CT performed within a 3-month window to minimize temporal confounding.

#### 2.2.2 Exclusion criteria

To minimize confounders affecting BMD or vascular health, we excluded patients with: (1) impairments preventing independent ambulation (e.g., recent major fracture, amputation); (2) significant skeletal deformities affecting DXA accuracy (e.g., severe kyphosis); (3) cognitive or psychiatric disorders hindering cooperation; (4) use of anti-osteoporotic medications within three months prior to the dual-energy X-ray absorptiometry scan; (5) concurrent diagnosis of active malignancy, severe chronic kidney disease (stage 4 or 5), thyroid or parathyroid dysfunction, or systemic rheumatic disease. (6) Uncontrolled diabetes mellitus (HbA1c > 10% or requiring insulin intensification at enrollment), given its established independent effects on both bone mineral density and vascular calcification.

### 2.3 CT imaging protocol

Non-contrast chest CT was performed examinations to employ a non-contrast protocol that is consistent and uniform. This protocol is implemented using a Philips Incisive 64-slice/128-slice CT scanner (Philips Healthcare, Amsterdam, the Netherlands). Patients are to be positioned supine with their arms raised overhead. Imaging is acquired during a single deep breath-hold. The scan range encompasses the entire thoracic cavity, extending from the lung apices to the diaphragm. The parameters for tube voltage (120 kV) and automatic tube current modulation (for dose optimisation) are applied. While standard thickness was set at 5.0 mm, images were reconstructed at 1.0 mm using a multi-plane reconstruction algorithm to facilitate AI-based analysis.

### 2.4 Quantitative analysis of CAC using AI

Non-contrast CT sequences were retrieved from the institution's PACS. Automated CAC quantification was performed using the U-Vision Intelligent Non-Gated CAC Scoring System (AI Discover Version R001, United Imaging Healthcare, Shanghai, China), which is an NMPA-cleared deep learning platform. The software executed a serial analysis workflow, which involved the automatic segmentation of cardiac structures and the subsequent application of standardized thresholds of attenuation values >130 Hounsfield units and dimensions ≥1 mm^2^. Finally, branch-specific Agatston scores are calculated for each vessel (10, 13).

**Figure 2:**
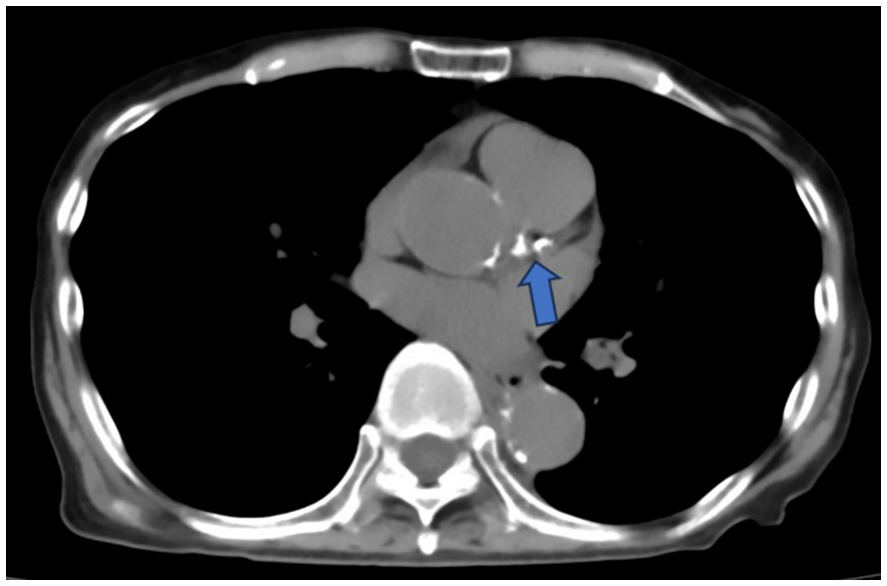
Non-contrast, nongated chest CT scan from an 88-year-old male with COPD and osteoporosis.

**Figure 3:**
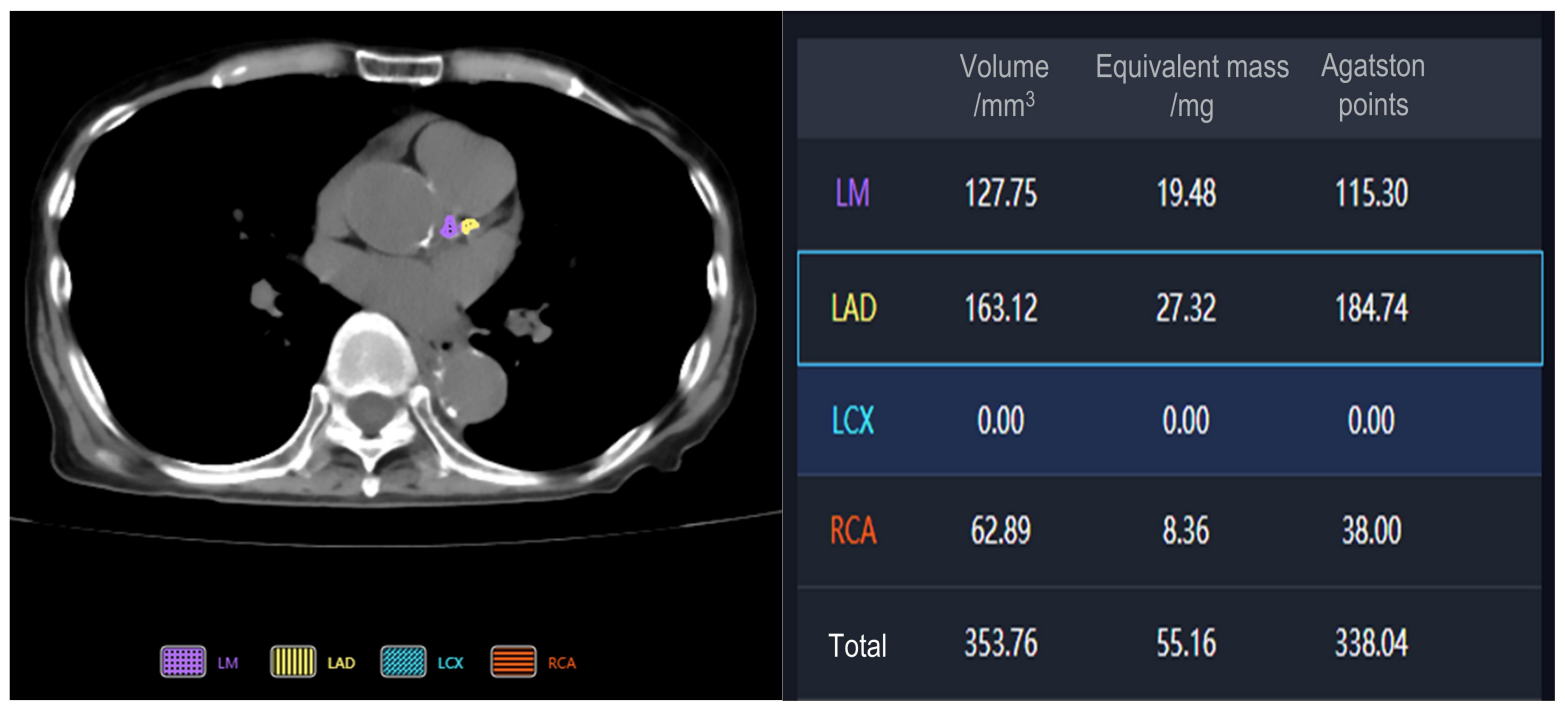
Automated AI quantification of coronary artery calcium (uAI Discover-Nongated Coronary Artery Calcium Score) on a nongated chest CT in an 88-year-old male with COPD and osteoporosis (Figure 2 shows the same patient).

**Figure 4:**
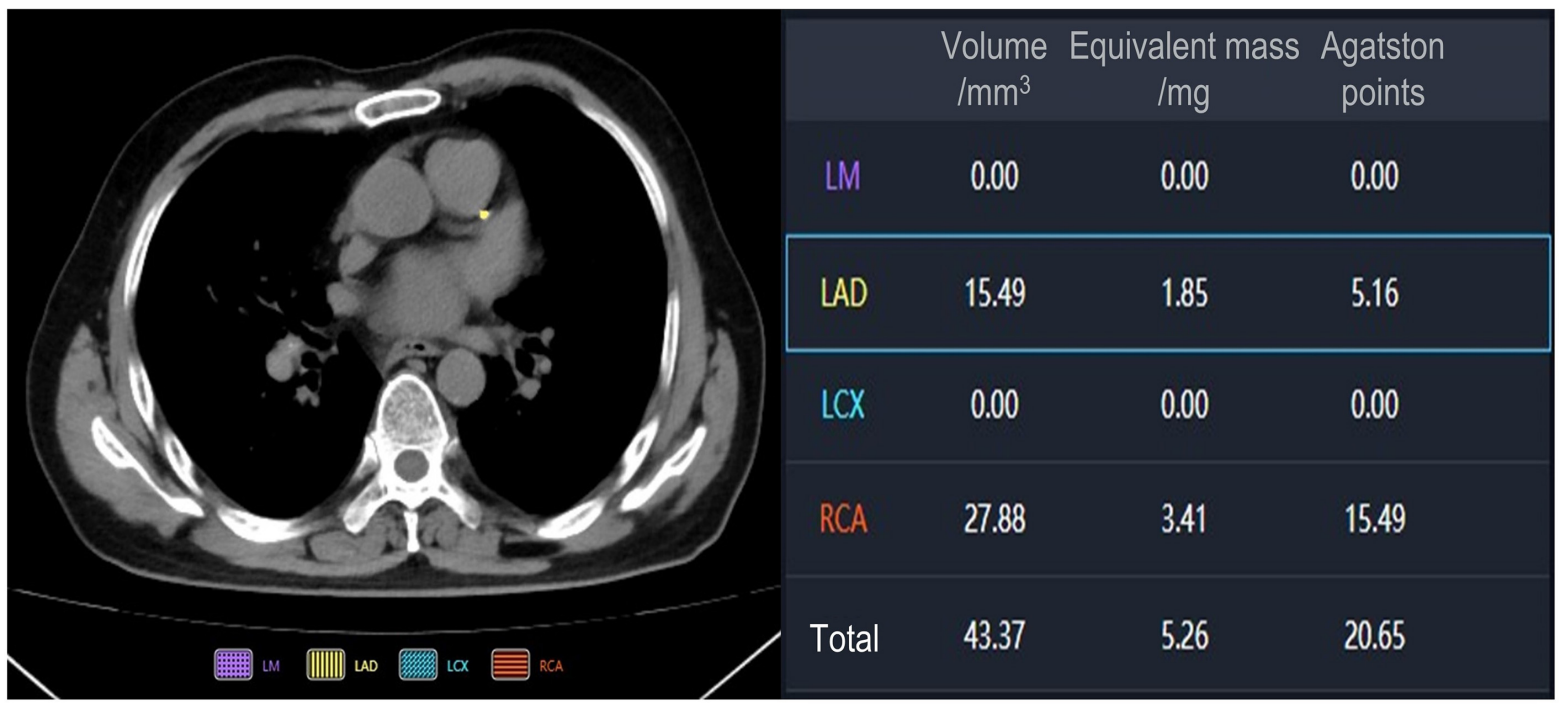
Automated AI quantification of coronary artery calcium (uAI Discover-Nongated Coronary Artery Calcium Score) on a nongated, non-contrast chest CT scan from a 66-year-old male with COPD but without osteoporosis.

To ensure precision, a two-tier quality assurance process was implemented. All scans were independently reviewed by two board-certified radiologists (each with >10 years of experience). If necessary, manual scoring was performed using a dedicated mediastinal window. Discrepancies were resolved by consensus involving a third senior radiologist (>16 years of experience). All quality assurance reviews and manual corrections were performed on 1.0 mm reconstructed images displayed at mediastinal window settings (Figures 2–4).

### 2.5 Ethics statement

Approval for this retrospective study was granted by the Ethics Committee of Affiliated Hospital of Putian University (Approval No.: PYL[202595]). The committee waived the requirement for individual informed consent for the analysis of anonymized data.

### 2.6 Statistical analysis

Statistical analyses were conducted with SPSS (Version 25.0, IBM Corp., Armonk, NY, USA). Normally distributed continuous data are expressed as mean ± standard deviation (mean ± SD), and non-normally distributed continuous variables as median and interquartile range [M (P_25_, P_75_)]. Categorical data are summarized as frequencies and percentages (%). Group comparisons were made using the independent samples *t*-test for normally distributed continuous variables, the Mann–Whitney *U* test for non-normally distributed continuous variables, and the chi-square test for categorical variables. Variables that reached significance in univariate analyses were subsequently entered into a multivariable binary logistic regression model to determine independent predictors of OP in COPD patients. Based on the final regression model, independent predictors were used to generate receiver operating characteristic (ROC) curves, and the area under the curve (AUC) was computed. A two-sided *P*-value < 0.05 was considered statistically significant.

Variables achieving significance in univariate analyses were entered into the multivariable model using backward conditional stepwise selection (removal criterion: *P* > 0.10). Variance inflation factors (VIFs) were computed for all covariates; VIF < 5.0 was considered acceptable. To assess the stability of the primary predictor (LAD-CAC) in the presence of potential biochemical confounders, a sensitivity analysis was performed constructing three nested logistic regression models: (1) a base model including age, sex, BMI, and LAD-Agatston score; (2) an extended model additionally incorporating serum calcium and serum phosphorus; and (3) a full sensitivity model further adding corticosteroid use status. The Akaike Information Criterion (AIC) was reported for each model. Additionally, 1,000-iteration bootstrap resampling (Harrell's optimism-correction method) (21) was performed to estimate optimism-corrected AUC values as internal validation.

## 3 Results

### 3.1 Baseline characteristics

The final cohort analyzed in this study comprised 262 eligible patients: 117 (44.7%) in the COPD with OP group and 145 (55.3%) in the COPD without OP group. Patient age ranged from 50 to 90 years. Baseline characteristics and radiographic features are summarized in Table 1. This comparison revealed statistically significant intergroup differences in several key variables. Patients diagnosed with osteoporosis exhibited a higher mean age in comparison to those not diagnosed with the condition. The mean body mass index (BMI) was found to be lower in the OP group. Regarding the major imaging biomarkers under consideration, Agatston CAC scores in all four major coronary arteries—the right coronary artery (RCA), the left main trunk (LM), the left anterior descending artery (LAD), and the left circumflex (LCX)—were significantly higher in the OP group (all *P* < 0.05).

**Table 1:** Baseline characteristics in COPD patients with and without osteoporosis.

Variable	Non-osteoporosis (*n* = 145)	Osteoporosis (*n* = 117)	*t*/*Z*/*χ^2^*	*P*-value
Age, mean (SD), y	66.92 ± 8.38	73.60 ± 8.42	−6.40	0.000
Sex (*n*, %)			1.48	0.225
Male	131(56.7%)	100(43.3%)		
Female	14(45.2%)	17(54.8%)		
BMI, mean (SD), kg/m^2^	22.75 ± 3.11	21.18 ± 3.62	3.79	0.000
LM-CAC[M(P_25_, P_75_)]	0(−0.48, 2.75)	0(6.62, 23.76)	−4.02	0.000
LAD-CAC[M(P_25_, P_75_)]	0(1.89, 8.81)	1.51(35.92, 133.40)	−6.95	0.000
LCX-CAC [M(P_25_, P_75_)]	0(0.14, 0.79)	0(6.46, 33.46)	−4.85	0.000
RCAC[M(P_25_, P_75_)]	0(6.50, 20.30)	0(27.50, 110.75)	−2.28	0.022

Values are presented as means ± standard deviations, n (%), or M (P_25_, P_75_).

Categorical variables are presented as numbers of patients, with percentages in parentheses.

BMI, body mass index; LM-CAC, left main trunk coronary artery calcification; LAD-CAC, left anterior descending coronary artery calcification; LCX-CAC, left circumflex coronary artery calcification; RCAC, right coronary artery calcification.

Serum biochemical indices also differed significantly between groups (Table 2). The osteoporosis group demonstrated significantly lower serum calcium (2.224 ± 0.138 vs. 2.290 +/– 0.184 mmol/L, *p* < 0.001) and lower serum phosphorus (0.983 ± 0.205 vs. 1.054 ± 0.209 mmol/L, *P* = 0.019) compared to the non-osteoporosis group, while both groups remained within normal reference ranges. Alkaline phosphatase did not differ significantly (81.8 ± 35.8 vs. 76.8 ± 28.7 U/L, *P* = 0.232). Corticosteroid use was significantly more prevalent in the osteoporosis group (45.5%, 51/112) than in the non-osteoporosis group (24.4%, 32/131; *P* = 0.001).

**Table 2:** Serum biochemistry and corticosteroid use—inter-group comparison.

Variable	COPD + osteoporosis (*n* = 116–118)	COPD without osteoporosis (*n* = 131–145)	*P*-value	Clinical note
Serum calcium (mmol/L), mean ± SD	2.224 ± 0.138	2.290 ± 0.184	**< 0.001** ^*^	Both groups within normal range (2.20–2.65 mmol/L); OP group at lower-normal boundary
Serum phosphorus (mmol/L), mean ± SD	0.983 ± 0.205	1.054 ± 0.209	**0.019** ^*^	Consistent with impaired Ca-P axis; normal range 0.81–1.45 mmol/L
Alkaline phosphatase (U/L), mean ± SD	81.8 ± 35.8	76.8 ± 28.7	0.232 (NS)	No significant difference in bone turnover marker
Blood glucose (mmol/L), mean ± SD	7.53 ± 4.31	7.00 ± 4.22	0.041	Borderline higher in OP group
**Corticosteroid use, n (%)**	**51/112 (45.5%)**	**32/131 (24.4%)**	**0.001** ^*^	Significantly more prevalent in OP group; confirmed independent confounder

Mann–Whitney U-test for continuous variables (non-normal distribution by Shapiro–Wilk); χ^2^ test for corticosteroid proportions. ^*^P < 0.05. Bold values indicate statistically significant results (*p* < 0.05).

### 3.2 Independent predictors of osteoporosis

To identify factors independently associated with osteoporosis, a multivariable binary logistic regression model was employed, adjusting for age, sex, and BMI (Table 3). The adjusted analysis revealed that calcium load in two specific coronary arteries served as significant independent predictor of osteoporosis status in the COPD cohort. Specifically, each unit increase in the LAD-CAC score was associated with an odds ratio (OR) of 1.01 (95% CI: 1.00–1.02; *P* = 0.036). Similarly, the OR for each unit increase in the LCX-CAC score was 1.11 (95% CI: 1.02–1.20; *P* = 0.021).

**Table 3:** Multivariate analysis of factors associated with osteoporosis in COPD patients.

Variable	β	S.E	Wald	*P*	OR	95% CI
Age	0.08	0.02	21.28	0.000	1.09	1.05–1.13
BMI	−0.14	0.05	8.46	0.004	0.87	0.80–0.96
LM-CAC	−0.01	0.01	0.42	0.515	0.99	0.96–1.02
LAD-CAC	0.01	0.04	4.40	0.036	1.01	1.00–1.02
LCX-CAC	0.10	0	5.37	0.021	1.11	1.02–1.20
RCAC	0	1.52	0.81	0.369	1.00	1.00–1.01

BMI, body mass index; LM-CAC, left main trunk coronary artery calcification; LAD-CAC, left anterior descending coronary artery calcification; LCX-CAC, left circumflex coronary artery calcification; RCAC, right coronary artery calcification; OR, odds ratio; CI, confidence interval.

### 3.3 Predictive performance

**Figure 5:**
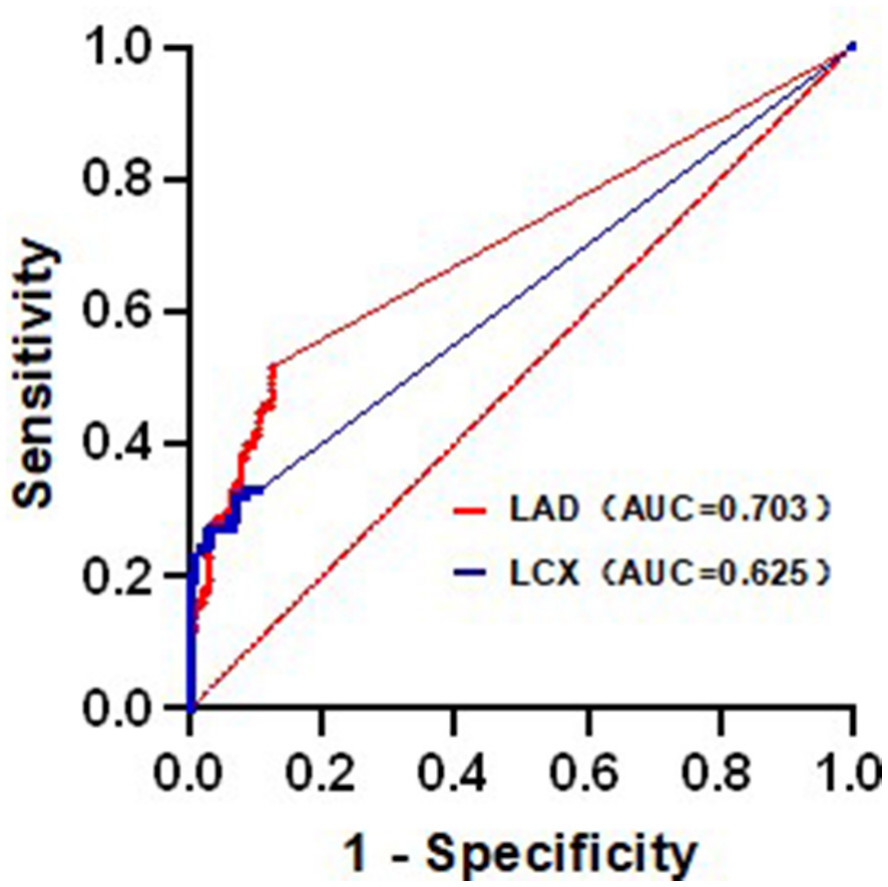
ROC Analysis of LAD-CAC and LCX-CAC for predicting osteoporosis in COPD patients. LAD-CAC, left anterior descending; LCX-CAC, left circumflex.

ROC curve analysis was performed to evaluate the discriminatory performance of these predictors. The AUC for the LAD-CAC score in predicting osteoporosis was 0.703 (95% CI: 0.638–0.769), while the AUC for the LCX-CAC score was 0.625 (95% CI: 0.556–0.695). The optimal cutoff values for each score, along with their corresponding sensitivity and specificity metrics (Table 4 and Figure 5).

**Table 4:** ROC curves of CAC as a significant predictor in the logistic regression model for COPD with osteoporosis.

CAC index	AUC(95%CI)	*P*	OCV	Sensitivity	Specificity	YI
LAD-CAC	0.703(0.638, 0.769)	0.000	0.085	0.513	0.876	0.389
LAC-CAC	0.625(0.556, 0.695)	0.001	0.885	0.316	0.931	0.247

ROC, receiver operating characteristic; AUC, area under the curve; CI, confidence interval; OCV, optimal cutoff value; YI, Youden index; CAC, coronary artery calcification; LAD-CAC, left anterior descending coronary artery calcification; LCX-CAC, left circumflex coronary artery calcification.

Bootstrap internal validation (1,000 iterations, Harrell's optimism-correction method) (21) yielded optimism-corrected AUC values of 0.779 (95% CI: 0.730–0.844) for LAD-CAC and 0.776 (95% CI: 0.729–0.842) for LCX-CAC, with mean optimism estimates of 0.009 and 0.008, respectively—confirming negligible model overfitting (Table 5). All 1,000/1,000 bootstrap iterations converged successfully.

**Table 5:** Bootstrap internal validation results (*B* = 1,000 iterations, Harrell optimism-correction method).

Model	*N* (events)	Apparent AUC	Mean optimism (95% CI)	Optimism-corrected AUC	Bootstrap 95% CI	Converged (out of 1,000)
**LAD-CAC model (age** **+** **sex** **+** **BMI** **+** **LAD)**	263 (118)	0.788	0.009 (−0.047–0.061)	**0.779**	0.730–0.844	1,000/1,000
**LCX-CAC model (age** **+** **sex** **+** **BMI** **+** **LCX)**	263 (118)	0.784	0.008 (−0.048–0.064)	**0.776**	0.729–0.842	1,000/1,000
Combined model (age + sex + BMI + LAD + LCX)	263 (118)	0.795	0.010 (−0.043–0.063)	0.784	0.740–0.848	1,000/1,000
Full model (all four CAC variables)	263 (118)	0.798	0.018 (−0.038–0.070)	0.779	0.745–0.848	1,000/1,000

AUC categories per Hosmer and Lemeshow: 0.7–0.8 = moderate; ≥0.8 = good. Negative lower bounds on optimism CI are expected and indicate true optimism near zero. EPV = 29.5 for single-predictor models. Bold values indicate statistically significant results (*p* < 0.05).

Sensitivity analysis further confirmed the robustness of the primary predictor. The adjusted OR for LAD-CAC remained virtually unchanged across all three nested model specifications: 1.025 (95% CI: 1.013–1.036) in the base model, 1.026 (95% CI: 1.014–1.038) after additional adjustment for serum calcium and phosphorus, and 1.027 (95% CI: 1.014–1.039) after further adjustment for corticosteroid use (all *p* < 0.001), confirming that the independent predictive value of LAD-CAC is not confounded by calcium-phosphorus dysregulation or corticosteroid exposure (Table 6). Corticosteroid use was identified as an independent predictor of osteoporosis in the full model (OR = 3.07, 95% CI: 1.47–6.38, *P* = 0.003). The progressive decrease in AIC from 252.7 to 223.2 confirmed improved model fit.

**Table 6:** Sensitivity analysis: stability of LAD-CAC prediction after adjusting for Ca, P, and corticosteroids.

Model	LAD-CAC OR (95% CI)	*P* for LAD-CAC	AIC	Additional finding
Base model (age + sex + BMI + LAD-CAC)	1.025 (1.013–1.036)	< 0.001	252.7	—
Extended model (+ serum Ca + serum P)	1.026 (1.014–1.038)	< 0.001	249.2	Serum P: OR = 0.191, *P* = 0.052 (borderline protective)
**Full sensitivity model (+** **serum Ca** **+** **P** **+** **corticosteroids)**	**1.027 (1.014–1.039)**	**< 0.001**	223.2	**Corticosteroid use: OR** **=** **3.07 [1.47–6.38]**, ***P*** **=** 0.003^*^

*N* = 229 with complete data for all variables. LAD-CAC OR is virtually unchanged across all three model specifications (1.025 → 1.026 → 1.027), confirming independence from calcium–phosphorus dysregulation and corticosteroid exposure. Bold values indicate statistically significant results (*p* < 0.05). ^*^P < 0.05.

## 4 Discussion

### 4.1 Summary of key findings

This study is the first to investigate the association between AI-derived, branch-specific CAC scores from routine non-gated chest CT using AI software and OP within a COPD patient population. The principal findings are as follows: (1) Patients with COPD and OP were older, had lower BMI, and exhibited significantly higher CAC Agatston scores in the LM, LAD, LCX, and RCA compared to the non-OP group; (2) LAD-CAC and LCX-CAC were identified as independent predictors of OP in COPD patients; (3) ROC curve analysis indicated that LAD-CAC and LCX-CAC possess moderate predictive capability for OP. These findings provide a novel and potentially practical approach for leveraging routine imaging in COPD patients to screen for OP risk.

### 4.2 Anatomical specificity and the bone-vascular axis

The present study corroborates a branch-specific association between vascular calcification and OP. Previous investigations of bone-vascular relationships have primarily focused on the correlation between bone mineral density and total CAC score or its presence. However, this methodology has yielded significant inconsistencies in the literature (3, 14). The present study's findings are supported by a more refined vascular-level analysis, which reveals that calcification within the LAD and LCX supply regions holds specific predictive significance. This anatomical specificity may be attributed to the distinct local environments of different coronary arteries, which are exposed to varying hemodynamic shear stress patterns, anatomical orientations, and inflammatory microenvironments (6, 15). The LAD artery is the primary conduit for antegrade myocardial perfusion. The LAD may serve as a sensitive sentinel of systemic metabolic dysregulation and chronic inflammation. These are pathological processes that are recognized as key drivers of accelerated bone resorption (2, 9). In comparison to approaches that rely exclusively on composite scores, an analysis of branch-level calcification offers a more nuanced perspective on the exploration of shared pathophysiological mechanisms between the cardiovascular and skeletal systems.

### 4.3 Methodological strengths: AI and opportunistic screening

From a methodological perspective, the application of deep learning-based AI algorithms to achieve automatic quantitative analysis of CAC in non-gated chest CT scans represents a key strength of this study. Unlike traditional ECG-gated protocols that require complex procedures and higher radiation exposure (5), our methodology aligns with evidence supporting the reliability of non-gated CAC assessment (13). The integration of AI automation has been shown to enhance objectivity and reproducibility, while mitigating the labor-intensive nature and inter-observer variability of manual scoring (16). This integrated workflow facilitates “opportunistic screening,” extracting prognostic OP risk data from routine respiratory imaging at zero additional cost or radiation. This approach addresses the clinical challenge of low OP screening rates in the high-risk population (4).

A recent study by Bauer et al. similarly demonstrated the utility of opportunistic chest CT-based bone density assessment in COPD patients, achieving AUC values of 0.72–0.78 for DXA-defined osteoporosis (20). While that approach targeted vertebral trabecular attenuation, our method uniquely captures the vascular-skeletal axis via automated branch-level CAC quantification—a complementary strategy that may provide additive prognostic value.

### 4.4 Shared pathophysiological mechanisms

The tripartite association between COPD, CAC, and OP is likely driven by interconnected biological mechanisms, with chronic systemic inflammation as the core link. Persistent local and systemic inflammation in COPD, characterized by elevated pro-inflammatory cytokines such as tumor necrosis factor-α (TNF-α) and interleukin-6 (IL-6), initiates multifocal damage (2, 7). These cytokines promote lung tissue destruction while simultaneously stimulating osteoclastogenesis and bone resorption. Concurrently, they trigger the phenotypic transition of vascular smooth muscle cells into osteoblast-like cells, accelerating vascular calcification (16). Furthermore, the presence of vitamin D deficiency and calcium-phosphorus imbalance, two pathological manifestations commonly observed in patients with COPD, has been demonstrated to not only impair bone mineralization but also create a favorable environment for ectopic vascular calcification by regulating endogenous inhibitory proteins. At the molecular level, the process is characterized by dysregulation of shared signaling pathways, including the RANK/RANKL/OPG axis and the Wnt/β-catenin cascade. These have been demonstrated to be associated with the pathogenesis of OP and vascular calcification, suggesting the presence of common mechanistic disturbances (17, 18). A series of coexisting nonspecific risk factors, including advanced age, reduced exercise, and frequent glucocorticoid use, may exert synergistic effects in COPD patients, accelerating atherosclerotic plaque progression and bone mineral loss (19). The observation that LAD and LCX calcification retain predictive value even after statistical adjustment for age and BMI further confirms that the influence of these shared pathological mechanisms extends beyond simple physiological aging or body composition.

In keeping with this mechanistic framework, our biochemical data demonstrate that COPD patients with osteoporosis exhibited significantly lower serum calcium (2.224 ± 0.138 mmol/L) and serum phosphorus (0.983 ± 0.205 mmol/L) compared to those without osteoporosis (*p* < 0.001 and *P* = 0.019, respectively), consistent with chronic vitamin D deficiency reducing intestinal calcium absorption and triggering compensatory secondary hyperparathyroidism. Corticosteroid use was significantly more prevalent in the osteoporosis group (45.5% vs. 24.4%, *P* = 0.001), confirming the contribution of glucocorticoid-induced osteoblast inhibition.

### 4.5 Clinical implications and limitations

Our findings offer a preliminary framework for clinical screening. For COPD patients undergoing routine CT monitoring, embedded AI-CAC analysis can automatically flag significant LAD or LCX calcification. These “alert markers” in radiology reports could prompt clinicians to pursue DXA confirmatory testing and initiate early preventive strategies, such as lifestyle optimization, fall prevention, or pharmacotherapy (2, 4). The implementation of such strategies has the potential to address the existing gaps in current screening practices, thereby reducing morbidity and mortality rates among patients with COPD due to osteoporotic fractures.

Sensitivity analyses incorporating serum calcium, serum phosphorus, and corticosteroid use as additional covariates confirmed the robustness of LAD-CAC as an independent predictor: its adjusted OR remained stable across all three model specifications (1.025–1.027, all *p* < 0.001), indicating that the CAC-osteoporosis association is not explained by calcium-phosphorus metabolic dysregulation or corticosteroid exposure. The identification of corticosteroid use as an independent risk factor (OR = 3.07, 95% CI: 1.47–6.38, *P* = 0.003) underscores the importance of routine osteoporosis screening in COPD patients receiving long-term corticosteroid therapy.

Despite these strengths, this study has limitations. Its retrospective, single-center design may limit external generalizability. While statistically powered for exploratory analysis, larger multicenter prospective cohorts are needed for validation. The cross-sectional nature precludes definitive causal inferences regarding the temporal relationship between CAC and bone loss. Additionally, we relied on DXA-derived areal BMD; future research utilizing quantitative CT (QCT) could provide deeper insights into volumetric density and trabecular microarchitecture (9). Finally, unmeasured confounders, such as detailed biochemical markers (e.g., vitamin D, PTH) and comprehensive medication histories, should be incorporated into future models to enhance predictive precision and further elucidate underlying mechanisms.

Several additional critical limitations must be acknowledged. First, the low proportion of female patients limits generalizability; sex-stratified analyses were underpowered. Second, chronic corticosteroid exposure was not quantified by cumulative dose; future studies should stratify by corticosteroid burden. Third, the deep learning AI system does not provide integrated explainability features (e.g., Grad-CAM, SHAP); interpretability frameworks are required before clinical deployment. Fourth, the limited sensitivity of CAC predictors (51.3% for LAD-CAC; 31.6% for LCX-CAC) precludes standalone screening use; they function as rule-in adjuncts to guide targeted DXA referral.

## 5 Conclusion

This study demonstrates that, in patients with COPD, the AI-derived calcium scores for the left anterior descending and left circumflex coronary arteries, automatically extracted from routine non-gated chest CT images, are independently associated with concomitant osteoporosis and offer significant predictive utility. This suggests that utilizing existing diagnostic chest CT images for automated CAC assessment provides a convenient, cost-effective, and “opportunistic” adjunctive screening tool to identify high-risk individuals within the COPD population. Future research should be dedicated to validating these results in prospective cohorts and further elucidating the shared inflammatory and molecular pathways linking COPD, CAC, and osteoporosis.

## Data Availability

The original contributions presented in the study are included in the article/supplementary material, further inquiries can be directed to the corresponding author.
